# Highlighting Survival with Yttrium-90 Radioembolization Therapy in Unresectable Hepatocellular Carcinoma

**DOI:** 10.7759/cureus.8163

**Published:** 2020-05-16

**Authors:** Edward Nabrinsky, Edward James

**Affiliations:** 1 Internal Medicine, Advocate Lutheran General Hospital, Park Ridge, USA; 2 Medical Oncology, Advocate Lutheran General Hospital, Park Ridge, USA

**Keywords:** yttrium-90, y90, hepatocellular carcinoma, hcc, sorafenib, immunotherapy, hepatology, gi, oncology

## Abstract

Unresectable hepatocellular carcinoma has several different therapeutic options, including targeted agents as well as locoregional therapy. Yttrium-90 (Y90) radioembolization therapy is an established treatment for unresectable disease and has been compared to other locoregional options as well as different targeted therapies. Newer case series are also reporting a potential benefit to the addition of immunotherapy to Y90 radioembolization. Here we report a case of prolonged survival in a patient whose treatment course included Y90 radioembolization along with sorafenib and nivolumab.

## Introduction

Hepatocellular carcinoma (HCC) is the most common form of primary liver cancer, affecting millions of people across the world [1]. While resectable disease is associated with positive survival trends, unresectable disease presents a challenge for containment using both locoregional therapies as well as targeted agents. Yttrium-90 (Y90) radioembolization therapy has been used since the middle of the decade for unresectable disease, and has had favorable outcomes and tolerability in comparison to transarterial chemoembolization (TACE) treatment [2]. However, no clear overall survival (OS) trends have been shown in comparison to targeted treatments. Several emerging case reports may demonstrate benefit when combined with immunotherapy [3,4]. We highlight a case of prolonged survival in a patient who received a combination of Y90 radioembolization therapy with sorafenib, transarterial chemoembolization as well as nivolumab.

## Case presentation

A 60-year-old male with past medical history notable for rheumatoid arthritis initially presented to the emergency department after abnormal outpatient blood work. He endorsed a drinking history several decades prior to presentation. Screening labs were significant for an aspartate aminotransferase of 132 units (U)/L (normal range: <38), alanine aminotransferase of 132 U/L (<64), alkaline phosphatase of 140 U/L (45-117), and albumin of 3.2 mg/dL (3.6-5.1), with normal total and direct bilirubin as well as normal total protein. Subsequent hepatitis panel demonstrated reactive hepatitis C antibody, with hepatitis C viral RNA by PCR of 601,466 U/L (<15).

**Figure 1:**
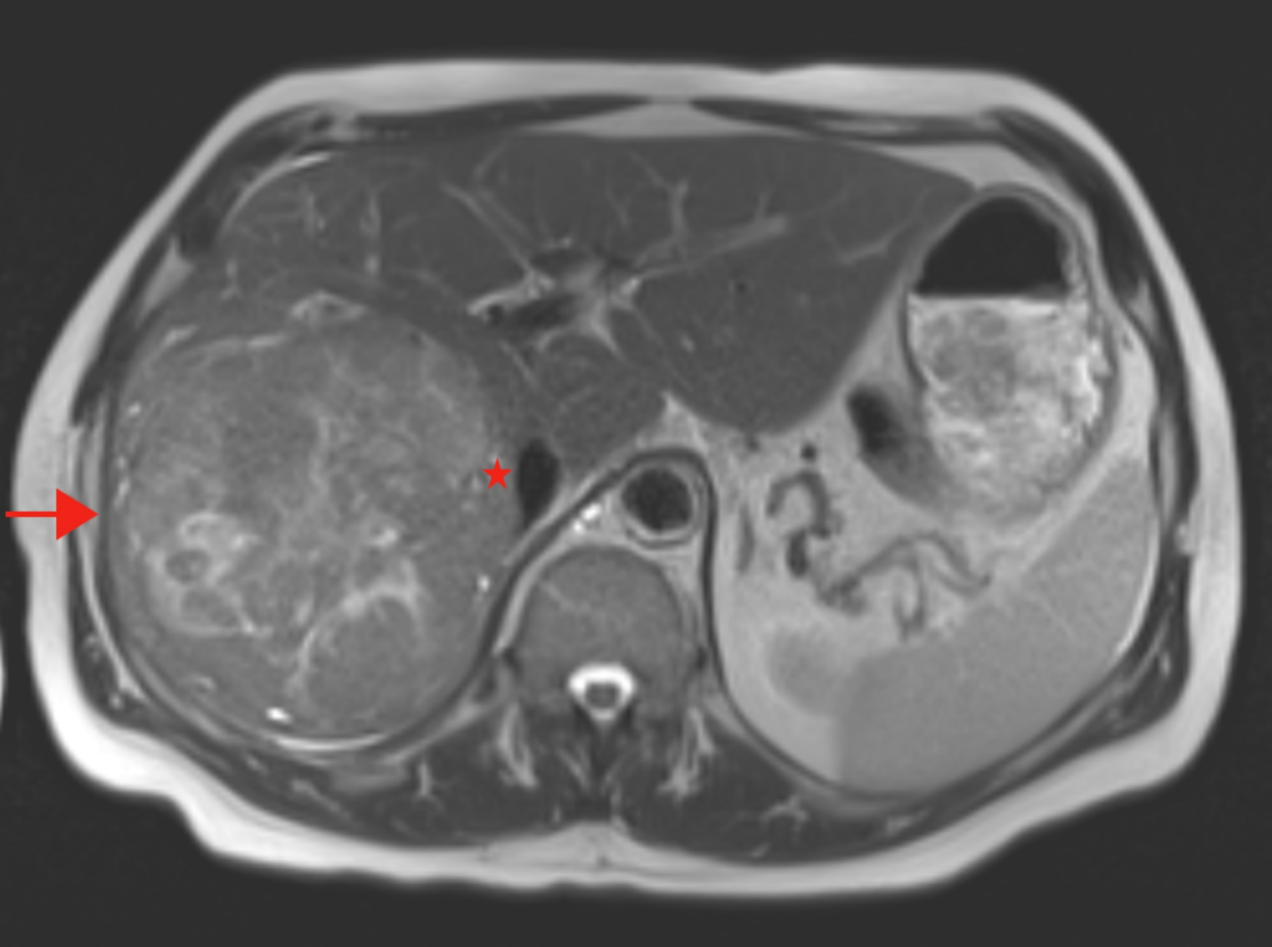
Presentation MRI of the abdomen A large heterogeneous mass in the right lobe of the liver is seen (arrow). Mild extension into the lateral wall of the intrahepatic inferior vena cava is also demonstrated (star).

The patient underwent liver ultrasound that demonstrated a mass involving the right hepatic lobe. Follow-up MRI was significant for a 11.1 x 11.3 x 11.7 cm heterogeneous mass in the right lobe of the liver, without nodular contour or cirrhotic morphology of the liver (Figure 1). Tumor extension into the right portal vein and main portal vein was noticed. Subsequent biopsy of the liver confirmed Stage IV A HCC, due to portal vein involvement. His alpha-fetoprotein (AFP) level at this time was 8 ng/mL (0-9). No evidence of extrahepatic spread was found on other imaging studies.

The patient was started on sorafenib twice per day after his diagnosis. He was not a candidate for transplantation due to having Stage IV A HCC, and TACE was contraindicated due to portal vein involvement. He then underwent Y90 radioembolization therapy three months after initial imaging via the right hepatic artery. He discontinued sorafenib seven months after diagnosis due to skin rash and abscesses requiring drainage.

**Figure 2:**
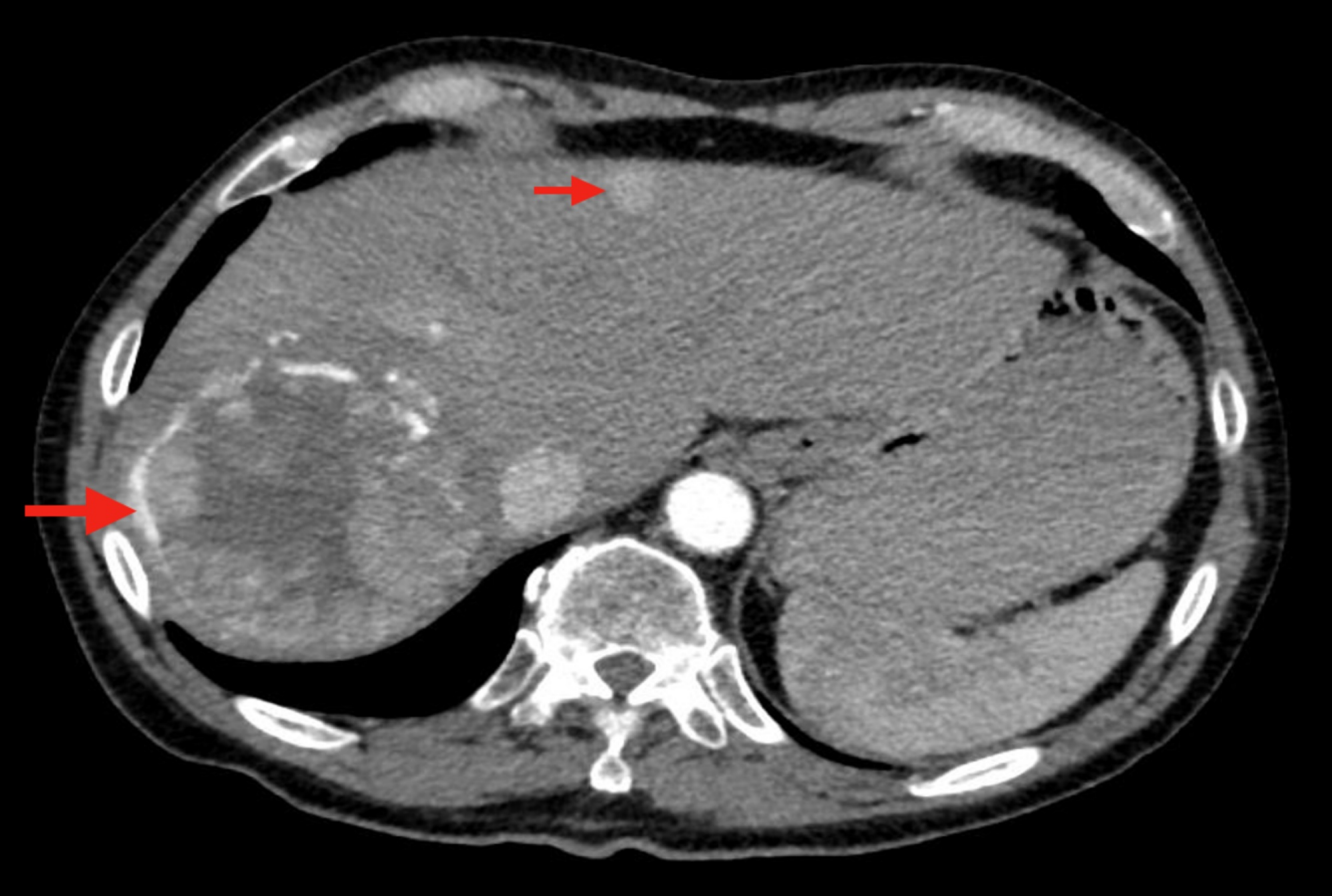
CT imaging 13 months after diagnosis The right hepatic heterogeneous mass (large arrow) demonstrates a central area of necrosis. The hepatic inferior vena cava does not appear to be invaded or compressed. A smaller lesion in the superior lobe of the left liver is also seen (small arrow).

CT imaging 13 months after diagnosis showed similar size of the right hepatic mass with a central area of necrosis, along with a new 13-mm lesion in the superior left lobe (Figure 2). The patient received doxorubicin chemoembolization to this left liver lesion two months later (15 months after diagnosis) with no additional intervention to the stable right-sided hepatic mass.

**Figure 3:**
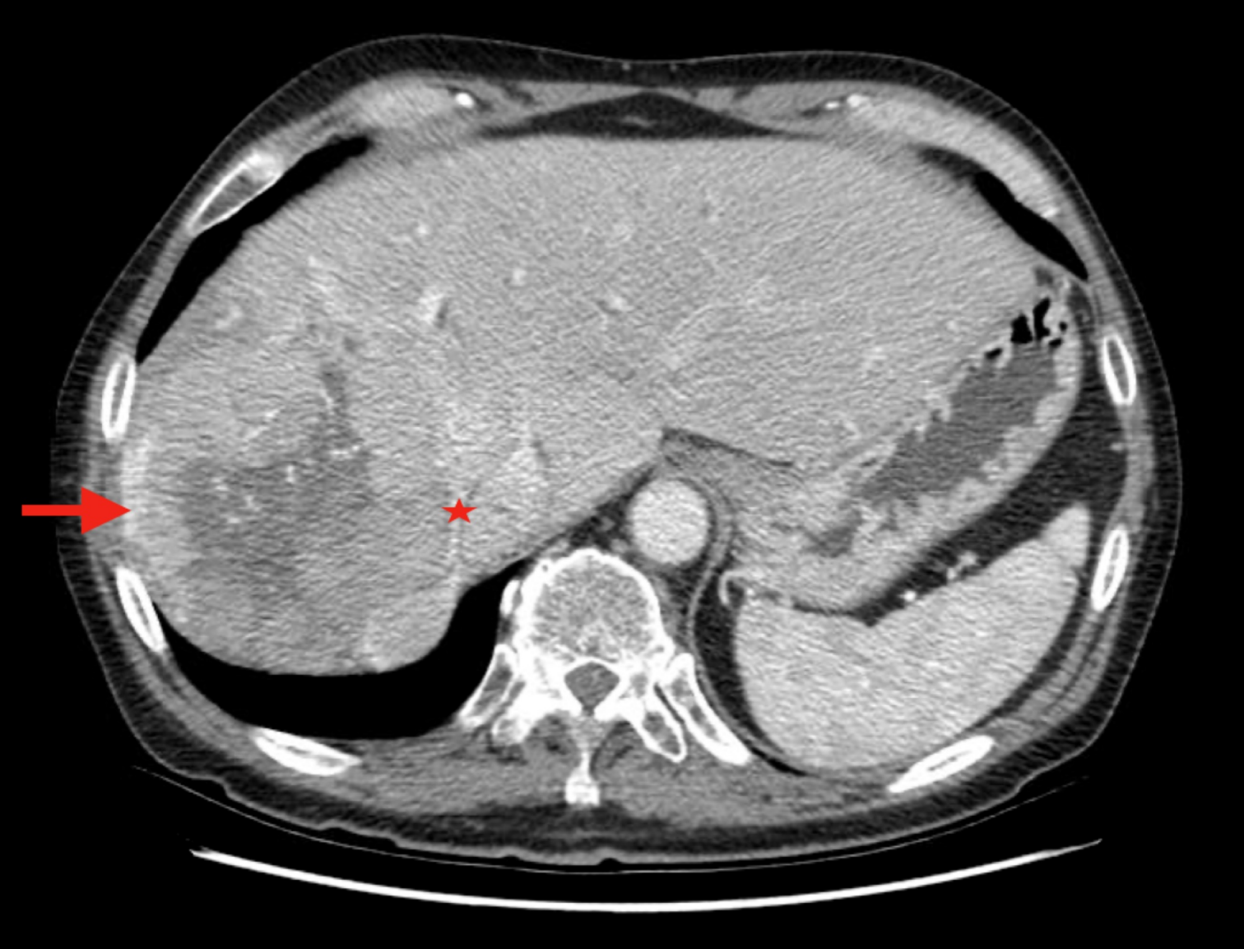
CT imaging 21 months after diagnosis Imaging continues to demonstrate a large right-sided heterogeneous mass (arrow), appearing larger than that in previous studies. The mass causes marked mass effect upon and narrowing of the inferior vena cava (star, medial to arrow).

Six months following the doxorubicin chemoembolization treatment (21 months after diagnosis), CT was significant for a diffusely enlarged liver compared to previous scans, with the right hepatic mass appearing larger and measuring approximately 19.0 x 14.1 x 15.3 cm (Figure 3). Calcification in the left lobe was stable, and tumor thrombus at the bifurcation of the main portal vein was appreciated, noted to be causing mass effect and narrowing of the inferior vena cava.

**Figure 4:**
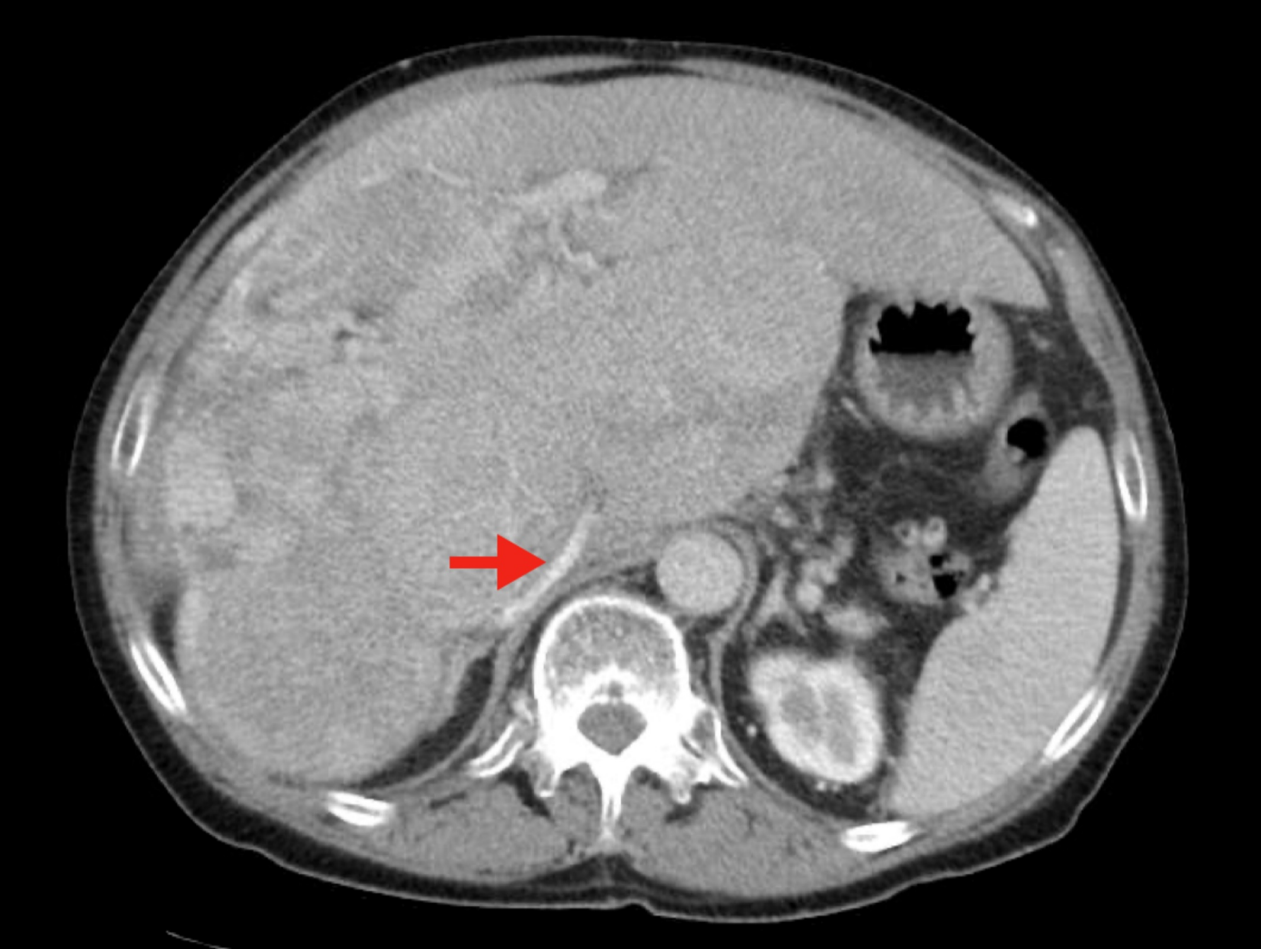
CT imaging 31 months after diagnosis demonstrating progression The liver is enlarged, with a persistent heterogeneous mass in the right hepatic lobe that is increased in size from previous studies. The mass causes mass effect and 'slitlike' appearance of the intrahepatic inferior vena cava (arrow).

His most recent surveillance CT 31 months after initial diagnosis demonstrates a continuously enlarging liver with right hepatic mass currently approximately 21.0 cm in greatest dimension, along with patchy areas of enhancement of the left hepatic lobe (Figure 4). The intrahepatic inferior vena remain compressed and appears slitlike.

Based on imaging studies, patient’s tumor’s time-to-progression (TTP) of the right hepatic lobe dominant mass was 21 months. His most recent AFP level was 34 ng/mL, up from 8 at the time of initial imaging studies. He was maintained on nivolumab immunotherapy for approximately 11 months; due to unfortunate insurance issues, he had treatment interruptions and passed away in October 2019.

## Discussion

HCC is the most common primary liver malignancy and a leading cause of cancer-related deaths across the world, ranking number 9 in the United States [1]. Both hepatitis B and C are independent risk factors for progression to HCC; when infected with hepatitis C, 80% of patients progress to chronic hepatitis while approximately 20% develop cirrhosis [5]. HCC in the context of hepatitis C occurs predominantly in the setting of cirrhosis. Other risk factors for HCC include alcohol consumption, tobacco use, diabetes mellitus, and occasionally genetics disease such as hemochromatosis, Wilson’s disease and alpha-1 antitrypsin deficiency [6]. Life expectancy in patients with HCC depends on the stage at diagnosis; while early-stage resectable tumors without vascular invasion can have a five-year survival rate approaching 50%-75%, survival rates for advanced disease are dismal [7].

Many different targeted molecular therapies have been developed for HCC, but the two most effective approaches are liver resection and transplantation, when possible [8]. In regard to targeted therapies, sorafenib, a monoclonal antibody directed against multiple targets including vascular endothelial growth factor (VEGF) receptor and Ras, was approved by the US Food and Drug Administration for advanced HCC after multiple phase II studies demonstrated benefit over placebo [5].

While well tolerated overall, side effects can include dermatologic toxicity, fatigue, hypertension, and hand-foot-mouth syndrome [4,5]. Other options for targeted therapies include other newer multikinase inhibitors such as lenvatinib, regorafenib, and cabozatinib, as well as other target agents such as bevacizumab, erlotinib, and cetuximab; inhibitors in the form of immunotherapy can also be considered for second-line treatment [6,8,9].

When resection and transplantation are not options, locoregional therapy can also be used in an attempt to control disease. This kind of therapy encompasses multiple treatments including radiofrequency ablation, transarterial radioembolization (TARE) with agents such as Y90, and TACE [5]. These treatments have developed in an attempt to eliminate the tumor’s blood supply via the hepatic artery, via particle embolization delivered in various modalities. Radiofrequency ablation is recommended for unresectable early stage patients, while both Y90 and TACE can be used for unresectable HCC.

In recent years, several studies have sought to compare the efficacy of Y90 radioembolization treatment to TACE in an attempt to define superior definitive treatment as well as to establish a role for downstaging cancer in an attempt to bridge to transplant [2]. We will focus on Y90’s radioembolization role in treatment, and patient outcomes with this modality.

Y90 radioembolization’s utility in HCC was first explored in the setting of portal vein thrombosis (PVT), as TACE with PVT is relatively contraindicated due to concern for iatrogenically induced acute liver failure [2]. This is important to note as anywhere from 10% to 40% of patients with HCC can have PVT at the time of diagnosis, while 35%-44% are found to have PVT at the time of death or liver transplant [10]. Because TARE is a local brachytherapy, it does not result in microvascular embolization and potential tumor ischemia [11]. In 2004, Salem et al. reported in a cohort of 15 patients with PVT that Y90 radioembolization was tolerated well with mild grade 1-2 bilirubin toxicity [12]. And so for the first time, Y90 radioembolization was considered safe in the setting of PVT. A more recent study also demonstrated that portal vein invasion does not affect survival in advanced stage HCC patients undergoing TARE with Y90 [8].

A meta-analysis by Zhang et al. in 2015 included eight studies with 1499 total patients, and compared TARE using Y90 to TACE in regard to safety and efficacy for unresectable HCC; they found that Y90 radioembolization was superior in regard to the OS, three-year OS rates, and TTP [13]. Specifically, there was a 26% reduction in risk of death for TARE patients (hazard ratio [HR] = 0.74, 95% CI 0.6-0.9) along with a statistically significant higher three-year OS rate compared to the TACE group (risk ratio [RR] = 1.75, 95% CI 1.03-3.03, p=0.05) [11]. The 2016 Prospective Randomized study of chEmoeMbolization versus radIoEmbolization for the tReatment of hEpatocellular carcinoma (PREMIERE), a phase II study comparing TTP in Barcelona Clinic Liver Cancer (BCLC) A and B patients, found a significantly longer TTP in TARE versus TACE, although it reported similar OS [14]. TACE can be more frequently associated with abdominal pain and increased transaminases [15].

Another aspect to examine is the predicted OS of patients with advanced-stage HCC. A meta-analysis by Rognoni et al. in 2016 identified 21 studies reporting data on OS and TTP with TARE treatment with Y90 in both intermediate and advanced stage HCC; the data suggested that median OS in patients receiving TARE for intermediate-advanced HCC with PVT was 6-12 months [11]. This was within the range of prior published studies reporting a median OS of 7-41.6 months for BCLC B and C patients [16].

It is also important to consider Y90 radioembolization in the context of sorafenib treatment. A 2014 phase I randomized trial taking an early look at Y90 radioembolization in combination with sorafenib did not demonstrate difference in survival rates between the two groups [7]. The 2018 Selective Internal Radiation Therapy Versus Sorafenib (SIRveNIB) trial, a phase III, randomized, multi-center study comparing sorafenib to Y90 radioembolization, demonstrated no statistically significant difference in median OS, but Y90 patients had higher TTP response rates and fewer adverse events [2,17]. These results were similar to 2017’s SorAfenib versus Radioembolization in Advanced Hepatocellular carcinoma (SARAH) trial, comparing Y90 radioembolization to sorafenib, where there were no survival benefits to Y90 [18].

We want to note that more recently, another promising treatment for first-line unresectable HCC has been investigated. The IMbrave150 trial’s intention-to-treat analysis evaluated 501 patients to receive either atezolizumab (or A), a humanized monoclonal antibody to programmed cell-death ligand 1, and bevacizumab (or B), a humanized monoclonal antibody to VEFG A (A+B, n=336), or sorafenib (n=165) in unresectable HCC with no prior therapy [19]. Coprimary endpoints of median OS and median progression-free survival (PFS) in the A+B group were not reached and 6.8 months, respectively, compared to 13.2 months and 4.3 months, respectively, in the sorafenib group. Patients also had more than twice the length of treatment duration (near 7.0 months for the A+B group compared to 2.8 for sorafenib), and grade ¾ adverse effects were similar in both groups [19]. This recent study highlights the potential role of frontline A+B in unresectable HCC, and the addition of Y90 to this therapy warrants evaluation in future trials.

Our patient highlighted the potential role of different therapies to work in combination with each other to provide a durable benefit in unresectable HCC. Similar to the 2014 phase I trial mentioned above, the 2018 SORAfenib in combination with local MICro-therapy guided by gadolinium-EOB-DTPA-enhanced MRI (SORAMIC) trial was a phase II study and evaluated sorafenib plus Y90 radioembolization versus sorafenib alone in 529 patients; while median OS rates were not very different, possible survival benefits were seen in patients younger than 65 as well as those without cirrhosis [2,20]. A 2019 case report from the University of Mississippi Medical Center reported a greater than one year response rate to consecutive treatment with TARE with Y90 followed by sorafenib and nivolumab in a patient with metastatic HCC on presentation [3]. This study also reported a case report describing a successful transition to partial hepatectomy from advanced HCC with combination Y90 radioembolization and nivolumab [4]. These studies raise the possibility of a benefit to combining standard Y90 radioembolization treatment with immunotherapy, possibly with the addition of sorafenib or other multikinase inhibitor treatment.

## Conclusions

Y90 radioembolization is an established treatment for unresectable HCC with PVT. Multiple studies have demonstrated benefit over TACE, although the survival benefit over sorafenib remains unclear at this point. Recent case reports demonstrate that there is a potential role for combination treatment with immunotherapy agents in combination with sorafenib. Our case highlighted that a single Y90 radioembolization treatment after sorafenib, later followed by TACE and immunotherapy, contributed to a continued prolonged survival for our patient. This highlights the need for larger trials comparing combination therapies for unresectable HCC, including evolving therapies with newer agents.
